# Optogenetic Termination of Cardiac Arrhythmia: Mechanistic Enlightenment and Therapeutic Application?

**DOI:** 10.3389/fphys.2019.00675

**Published:** 2019-06-06

**Authors:** Philipp Sasse, Maximilian Funken, Thomas Beiert, Tobias Bruegmann

**Affiliations:** ^1^ Institute of Physiology I, Medical Faculty, University of Bonn, Bonn, Germany; ^2^ Department of Internal Medicine II, University Hospital Bonn, University of Bonn, Bonn, Germany; ^3^ Research Training Group 1873, University of Bonn, Bonn, Germany; ^4^ Institute of Cardiovascular Physiology, University Medical Center, Georg August University Göttingen, Göttingen, Germany; ^5^ German Center for Cardiovascular Research (DZHK), Partner site Göttingen, Göttingen, Germany

**Keywords:** optogenetics, defibrillation, cardioversion, ventricular arrhythmia, ventricular tachycardia, ventricular fibrillation, atrial fibrillation, implantable cardioverter defibrillator

## Abstract

Optogenetic methods enable selective de- and hyperpolarization of cardiomyocytes expressing light-sensitive proteins within the myocardium. By using light, this technology provides very high spatial and temporal precision, which is in clear contrast to electrical stimulation. In addition, cardiomyocyte-specific expression would allow pain-free stimulation. In light of these intrinsic technical advantages, optogenetic methods provide an intriguing opportunity to understand and improve current strategies to terminate cardiac arrhythmia as well as for possible pain-free arrhythmia termination in patients in the future. In this review, we give a concise introduction to optogenetic stimulation of cardiomyocytes and the whole heart and summarize the recent progress on optogenetic defibrillation and cardioversion to terminate cardiac arrhythmia. Toward this aim, we specifically focus on the different mechanisms of optogenetic arrhythmia termination and how these might influence the prerequisites for success. Furthermore, we critically discuss the clinical perspectives and potential patient populations, which might benefit from optogenetic defibrillation devices.

## Introduction

### Optogenetic Tools Relevant to Cardiac Defibrillation

Optogenetics is a technology that employs light-sensitive proteins for the stimulation of cells and organs by illumination and can be used *in vitro* as well as *in vivo* (Hegemann and Nagel, 2013). Optogenetic stimulation provides unprecedented spatiotemporal resolution since light can be focused to specific regions using lenses or light fibers, and the exact time of activation and deactivation is defined by onset and duration of the illumination. Furthermore, by expressing optogenetic proteins under the control of cell type-specific promoters, the stimulation can be restricted to certain cell types of interest within an intact organ. The most frequently used optogenetic protein is Channelrhodopsin2 (ChR2), a light-gated, non-selective cation channel derived from green algae (Nagel et al., 2003). This protein with seven transmembrane domains contains all-trans-retinal as chromophore in which photon absorption leads to isomerization to the 13-cis form and subsequently opening of the channel pore, which mainly conducts Na^+^ and K^+^ ions. Fortunately, retinal as co-factor is already present in most mammalian tissues *in vivo* (Kane et al., 2005). Within excitable cells, ChR2 activation leads to inward currents and cell membrane depolarization, which allows contact-free control of the membrane potential. A myriad of different Channelrhodopsin variants have been created by amino acid exchanges, generating chimeric proteins or identifying new versions in other species in nature, which vary in their light sensitivity, wavelength specificity, and on- and off kinetics (Mattis et al., 2011). Besides channelrhodopsins, light-driven H^+^ and Cl^−^ pumps from bacteria and fungi were used to export H^+^ ions or import Cl^−^ ions, which leads to light-induced hyperpolarization and inhibition of electrical activity (Zhang et al., 2007; Mattis et al., 2011). New Cl^−^ selective ChR2 variants (Berndt et al., 2014; Wietek et al., 2014) as well as natural anion conducting light-gated channels (Govorunova et al., 2015) can also hyperpolarize some cell types; however, this depends strongly on the Nernst potential for Cl^−^. In fact, in cardiomyocytes, light-induced activation of anion-conducting channels at resting membrane potential leads to depolarization as shown elegantly by Kopton et al. in this Research Topic article collection (Kopton et al., 2018). Recently, K^+^ selective light-gated ion channels have been described for optogenetic silencing of electrical activity but with very slow off kinetics (Alberio et al., 2018; Bernal Sierra et al., 2018).

### Optogenetic Pacing of Cardiomyocytes and Intact Hearts

Ever since its development, optogenetics has been used in the field of neuroscience to study basic neurophysiology and disease mechanisms by light-induced modulation of electrical activity in neurons (Adesnik, 2018). Our group has shown for the first time that optogenetics can be employed for light-based pacing of cardiomyocytes *in vitro* and of the atria and ventricles of transgenic mice *in vivo* (Bruegmann et al., 2010). Similarly, Arrenberg and colleagues demonstrated in embryonic zebrafish hearts light-induced hyperpolarization with a light-driven Cl^−^ pump to block excitation as well as ChR2-based pacing (Arrenberg et al., 2010). These publications laid the foundation for the field of cardiac optogenetics with subsequent publications focusing mainly on technological developments and optogenetic pacemaking (Jia et al., 2011; Abilez, 2012; Boyle et al., 2013; Nussinovitch et al., 2014; Zaglia et al., 2015; Klimas et al., 2016; Lapp et al., 2017; Rehnelt et al., 2017). In addition to direct pacing by light-induced depolarization, modulation of intrinsic pacemaking mechanisms and induction of arrhythmic beating were performed by optogenetic stimulation of the G_q_ or G_s_ protein-coupled receptors Melanopsin (Beiert et al., 2014) or Jellyfish opsin (Makowka et al., 2019), respectively. To be able to propose clinical applications of cardiac optogenetics, our group has demonstrated that systemic injection of adeno-associated viruses (AAV) results in ChR2 expression in the mouse heart, which is sufficient for optogenetic pacing (Vogt et al., 2015). The AAV ChR2 gene transfer strategy was extended to rats in order to suggest optogenetic cardiac resynchronization by multi-point illumination (Nussinovitch and Gepstein, 2015). Proving the ability to express ChR2 in wild-type hearts, these papers allowed for the first time a translational perspective for cardiac optogenetics. Because the potential application of optogenetic pacing of the heart has been reviewed extensively (Entcheva, 2013, 2014, 2015; Ambrosi et al., 2014; Boyle et al., 2014, 2015; Klimas and Entcheva, 2014) and optogenetics would probably provide only minor advantage over implantable electrical pacemakers, we will focus in this review on optogenetic termination of cardiac arrhythmia.

## Optogenetic Termination of Cardiac Arrhythmia

### Cardiac Arrhythmia

Ventricular tachycardia (VT) and the subsequent degeneration into ventricular fibrillation (VF) are life-threatening arrhythmic states of the heart. These may result in a drop in cardiac output, reduction of arterial blood pressure, syncope, and often in sudden cardiac death. VT and VF occur after myocardial infarction, myocarditis, in patients with reduced ejection fraction, during electrolyte imbalance or because of side effects of drugs and mutations of cardiac ion channels. The primary therapy of these acutely life-threatening arrhythmias is defibrillation by an electric shock to resynchronize the heart. Atrial fibrillation (AF) represents the most common arrhythmia with growing incidence and is accompanied by an increase in morbidity and mortality despite being not acutely life-threatening (Lip et al., 2012). In early stages of AF, the first clinical aim is to restore sinus rhythm by cardioversion with external electrical shocks (Lip et al., 2012; Kirchhof et al., 2016).

**Figure 1:**
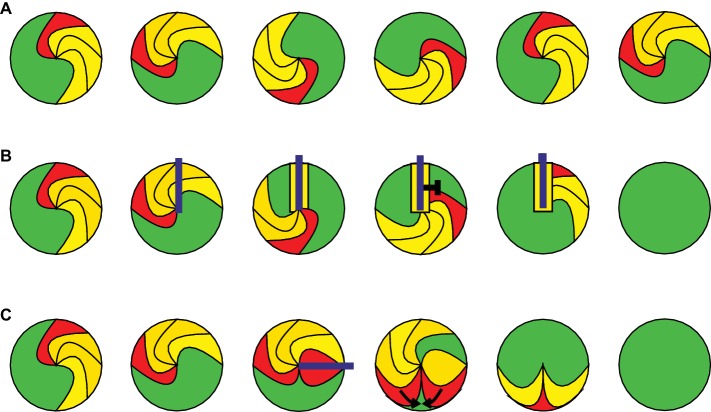
Mechanisms of cardiac arrhythmia and optogenetic termination. The leading edge of the arrhythmic wave front is shown in red, the depolarized and refractory tissue in yellow, and the excitable gap in green. The illumination is displayed in blue. **(A)** A stable rotor of a cardiac arrhythmia is shown at different time points during one cycle around a phase singularity. **(B)** Example for optogenetic arrhythmia termination by conduction block. Sustained illumination starts within a depolarized area and keeps the surrounding area depolarized until the next arrhythmic wave front entering this region is blocked by refractory tissue. **(C)** Optogenetic arrhythmia termination by filling of the excitable gap. Brief illumination of the excitable gap generates a second excitation wave front, which travels toward the arrhythmic wave front until both are extinguished by their collision.

Cardiac arrhythmias are usually initiated by an ectopic trigger and sustained by areas of slow conduction that promote the development of a so-called re-entry mechanism or the formation of rotors. Triggering occurs mainly during the diastole because of spontaneous opening of ion channels or Ca^2+^ release from intracellular stores. A re-entry mechanism is initiated, when the premature propagating wave front has to travel around a non-excitable region. Such regions can be non-excitable due to the anatomical structure (e.g., scar tissue) leading to re-entry wave fronts around the scar. Furthermore, electrophysiological heterogeneities such as partial refractory tissue (Wagner et al., 2015) can lead to cardiac electric rotors (Pandit and Jalife, 2013). A single macroscopic stable rotor (Figure 1A) or a re-entry wave front results in a monomorphic shape in the ECG. Additional rotors can be generated when waves traveling from the primary rotor encounter additional wavebreaks. This results in generation of highly periodic three-dimensional rotors that interact with each other in complex spatiotemporal patterns, which can be observed in the ECG as AF, polymorphic VT, or VF (Wagner et al., 2015).

### Mechanisms of Electrical Defibrillation

Although the risk of VT can be reduced by pharmacological treatment or catheter-based ablation of pro-arrhythmogenic regions in the ventricles, ventricular arrhythmias often re-occur in patients resulting in a high risk of sudden cardiac death. For this reason, patients with a propensity for VT/VF receive implantable cardiac defibrillators (ICD), which can terminate the arrhythmia by applying rapid antitachycardia pacing or finally by delivering a strong and painful electrical shock (Adgey et al., 2005). Even though the exact cellular mechanisms of defibrillation are still not fully understood, it was shown that it can be achieved by depolarization of a sufficient number of cells in accordance to the “critical mass theory.” This model was developed in the early 1970s when Doug Zipes and colleagues demonstrated that injection of K^+^ into the coronary arteries was able to defibrillate the canine heart (Zipes et al., 1975) probably by keeping cardiomyocytes depolarized and refractory and thereby block conduction. However, electrical shocks are restricted to few milliseconds and conduction block is unlikely to be sustained during electrical cardioversion or defibrillation. More likely, electrical shocks induce brief depolarization, which terminates arrhythmia by a mechanism called “filling of the excitable gap.” The excitable gap is the excitable myocardium (Figure 1A, green) between the trailing edge of the bypassed and the leading edge of the next reentrant wave front (Kleber and Rudy, 2004) and can be activated by the electrical shock or by antitachycardia pacing protocols producing a second wave front which will collide with the arrhythmic wave front. The effectiveness of this mechanism has been documented in clinical trials (De Maria et al., 2017) and depends on the duration and location of the excitable gap relative to the pacing site, which is rather difficult to predict. The excitable gap can also be reduced by increasing the cardiac wavelength [conduction velocity × action potential duration (APD)]. This is considered to be one alternative mechanism of electrical defibrillation because it has been shown that electrical shocks can temporally increase APD (Dillon, 1991).

Interestingly, also hyperpolarized areas occur in the cardiac tissue during an electrical shock because of the presence of virtual anodes. In fact the hyperpolarized myocardium is even larger in extent than depolarized tissue, because of asymmetric non-monotonic influence of the electric shock on myocyte membrane potential (Nikolski et al., 2004; Dosdall et al., 2010). In consequence, cardiac excitation can be initiated *de novo* at the boundaries of a virtual anode potentially causing re-initiation of a re-entry mechanism and failure of defibrillation (Efimov et al., 1998). Mathematical modeling of the ventricles suggests that such re-initiation could be prevented if hyperpolarization is strong enough to recover all Na^+^ channels from inactivation allowing the *de novo* excitations to travel fast enough through the virtual anode tissue to collide with the refractory tissue of depolarized areas (Cheng et al., 1998; Dosdall et al., 2010).

### *In vitro* Experiments on Cardiomyocyte Monolayers Predict Optogenetic Defibrillation

The idea of optogenetic arrhythmia termination (Knollmann, 2010) was generated by our early experiments *in vitro*, which showed that continuous illumination prolongs APD and refractory period of ChR2 expressing cardiomyocytes. Specifically, high intensity illumination led to a constant membrane potential above −35 mV, which keeps Na^+^ and Ca^2+^ channels inactivated. Consequently, we showed that in a monolayer of cardiomyocytes constant illumination was able to block electrical excitation and wave propagation into the illuminated region (Bruegmann et al., 2010), which led to the assumption that such conduction blocks can also be used to block arrhythmic wave fronts in the intact heart. This idea was supported by subsequent *in vitro* experiments by others with cardiomyocyte monolayers: Bingen and colleagues were able to show that low light intensity illumination reduces the excitability, slows conduction, and thereby terminates rotor wave fronts in a monolayer of atrial neonatal rat cardiomyocytes (Bingen et al., 2014). Furthermore, the groups of Gil Bub and Emilia Entcheva demonstrated that specific illumination patterns can be used to inscribe excitation waves *in vitro* in cardiomyocyte monolayers to control the direction of conduction and to destabilize arrhythmic excitation patterns (Burton et al., 2015). The concept was extended by real time optogenetic manipulation of the core of spiral waves allowing their attraction, anchoring and unpinning (“spiral wave dragging”) through controlled displacement of heterogeneities in atrial cardiomyocyte monolayers *in vitro* (Majumder et al., 2018). If these approaches using gentle modulation of cardiac arrhythmia patterns for self-extinction of planar waves in two-dimensional monolayers have an impact on scroll waves in the three-dimensional heart remains to be proven. Importantly, cores of scroll waves in the intact heart are represented as intramyocardial vortex filaments reaching from the epicardium to the endocardium, which has been recently visualized using high-resolution four-dimensional ultrasound-based strain imaging (Christoph et al., 2018). Thus “spiral wave dragging” of curved and non-perpendicular transmural vortex filaments with optogenetic approaches (“scroll wave dragging”) seems to be very challenging because of the technical challenge to bend light rays with high temporal and spatial flexibility.

### Optogenetic Defibrillation Using Conduction Block by Continuous Depolarization

One major advantage of optogenetic compared to electrical stimulation is the ability of constant depolarization by continuous illumination, which locks the illuminated region in absolute refractoriness and prevents (re-)excitation. Such conduction block would extinguish the arrhythmic wave front if conduction through the illuminated region was essential for arrhythmia maintenance (Figure 1B). However, in contrast to optogenetic pacing, which requires only epicardial excitation, effective conduction block would require sufficient epicardial illumination for transmural depolarization to inhibit mid-myocardial and endocardial wave propagation. About 6 years after establishing optogenetic pacing of mouse hearts, our group demonstrated optogenetic defibrillation in intact mouse hearts using constant illumination from the epicardial site, which terminated ventricular arrhythmia with a success rate of over 90% (Bruegmann et al., 2016). The main challenge was to find experimental conditions allowing stable ventricular arrhythmia without self-termination, which is difficult in the small mouse heart. We solved this by pharmacological opening of K_ATP_ channels to reduce APD and lowering the extracellular K^+^ concentration to slow conduction, which both shorten the cardiac wavelength to fit into the small mouse heart (Bruegmann et al., 2018). For successful defibrillation, sufficient light intensity as well as size of illumination was important, suggesting that transmural depolarization of the whole myocardial wall is essential in concordance with the results from the critical mass theory (Zipes et al., 1975). Furthermore, computational simulations of an infarcted patient heart expressing ChR2 *in silico* by the group of Natalia Trajanova helped to understand that sufficient transmural depolarization, including endocardial depolarization, to keep Na^+^ channels refractory is key to terminate a ventricular arrhythmia by epicardial illumination (Bruegmann et al., 2016). Similarly, experiments in ventricular slices from rat hearts (Watanabe et al., 2017) and in monolayers from neonatal rat cardiomyocytes revealed that arrhythmia termination in these two-dimensional systems requires illumination of one line spanning from the core region to the adjacent unexcitable parts (Feola et al., 2017). This would correlate to the required transmural depolarization in a three-dimensional heart to avoid endocardial wave front propagation. Our simulations showed that by epicardial illumination this condition can probably only be achieved using red light (669 nm) sensitive Channelrhodopsin variants because of the low penetrance of blue light (488 nm) in the thick human ventricular myocardium (Bruegmann et al., 2016).

Transmural depolarization to induce conduction block seems to be less challenging in the atria, because of the much thinner myocardial walls. In consequence, optogenetic cardioversion of atrial arrhythmia was successful in a ChR2 expressing mouse model heterozygous for an AF-promoting connexin 40/Gja5 mutation. This mouse line allowed induction of sustained AF episodes by intra-atrial electrical burst stimulation, which could be terminated by epicardial illumination (Bruegmann et al., 2018). Optogenetic AF termination was also proven in rats during muscarinic receptor stimulation to mimic vagal AF (Nyns et al., 2019). Because in both cases AF termination rates were highest at pulse duration >30 ms (Bruegmann et al., 2018) or >100 ms (Nyns et al., 2019), which is longer than the AF cycle time, it can be speculated that localized transmural conduction block and not filling of the excitable gap is the underlying mechanism.

### Defibrillation Using Filling of the Excitable Gap by Optogenetic Pacing

In contrast to creating a conduction block by continuous transmural optogenetic depolarization, filling of the excitable gap requires only brief (~few ms) depolarization of epicardial cardiomyocytes above the action potential threshold. In consequence, required light energy (intensity × duration) would be much lower and similar to optogenetic cardiac pacemaking. This concept was proven by the group of Leonardo Sacconi using patterned light stimulation to terminate VT with one specific reentrant wave front (Crocini et al., 2016). The authors did not use constant illumination but repetitive short light pulses to confined regions, which eventually activate the excitable gap creating a new wave front that collides with the arrhythmic wave front (Figure 1C). However, similar to electrical antitachycardia pacing, successful defibrillation using localized stimulation requires to know the extent of the excitable gap in time and space, otherwise the local conduction block mechanism would be more effective. We have demonstrated this recently in optogenetic AF termination by comparing the light intensities required for atrial pacing to those required for complete block of local electrical activity. AF termination efficacy was only ~50% using pacing light intensities, most likely because of random failure of stimulating the excitable gap. In contrast, application of higher light intensities, which are able to block electrical activity, terminated AF in all cases (Bruegmann et al., 2018).

The need for identification of the excitable gap in time and space can be circumvented using global pulsed illumination of the whole heart, which was shown in computer simulations on a human heart *in silico* (Karathanos et al., 2016). Furthermore, within this Research Topic article collection, Richter and colleagues showed experimentally on intact mouse hearts that indeed light pulses of low light intensity are sufficient for VT termination if ventricles are globally illuminated from all sites (Quinonez Uribe et al., 2018).

Because global homogeneous epicardial illumination of the human heart will be challenging, it will be important to compare defibrillation effectiveness by localized filling of the (previously identified) excitable gap using brief light pulses with localized conduction blocks by sustained illumination. Experimentally, this could be enabled by the recent development of an all-optical heart platform, combining epicardial voltage mapping with spatially defined optogenetic stimulation using a digital-mirror device in a closed-loop feedback system (Scardigli et al., 2018). Such a system would allow the on-line identification of reentrant wave fronts with excitable gaps and subsequent real-time illumination of the leading or trailing edge (Figure 1) as well as the rotor cores for potential “spiral/scroll wave dragging” (Majumder et al., 2018).

### Defibrillation by Optogenetic Hyperpolarization

The interplay between de- and hyperpolarized areas during defibrillation as well as specific effects of hyperpolarization alone cannot be experimentally addressed by electrical shocks with non-controllable (virtual) anodes and cathodes. In contrast, optogenetic methods allow selective hyperpolarization using light-driven H^+^ or Cl^−^ pumps. Within this Research Topic article collection, we report an optogenetic strategy to analyze the effects of hyperpolarization within the intact heart and to determine the potential mechanism for defibrillation (Funken et al., 2019). By expressing the light-inducible proton pump ArchaerhodopsinT in cardiomyocytes of transgenic mice, we were able to prove that hyperpolarization *per se* can terminate VA. Importantly, we identified a completely new VA termination mechanism by enhancing the electrical sink of the excitable gap presumably leading to conduction failure of high frequency wavelets with weak electrical source (source-sink mismatch). Unfortunately, the overall success rate was lower compared to conduction block by continuous depolarization with ChR2, which can be explained by the low efficiency of light-driven pumps as well as by simultaneous VA stabilizing mechanisms of hyperpolarization (increased Na^+^ channel availability resulting in enhanced electrical source of the arrhythmic wave front). For future clinical perspectives, more effective optogenetic tools for hyperpolarization would be necessary. Unfortunately, the recently presented K^+^ channel-based optogenetic approaches have very slow kinetics in the range of minutes (Alberio et al., 2018; Bernal Sierra et al., 2018). This would result in prolonged silencing of the ventricular activity even after defibrillation has occurred without reestablishment of blood circulation.

## Clinical Implications of Optogenetic Defibrillation

### Characteristics of Different Cardiac Arrhythmia Termination Mechanisms

In summary, we have identified two possible mechanisms for optogenetic termination of VA that can now be put into clinical context and be compared with current treatment strategies. (1) Optogenetic pacing to fill the excitable gap could be used for energy-reduced arrhythmia termination but requires either global illumination (Quinonez Uribe et al., 2018) or triggered localized illumination after mapping of the excitable gap by epicardial electrograms. (2) Generating a transmural conduction block by continuous optogenetic depolarization requires more light energy but only in predefined anatomical regions that are essential for arrhythmia re-entry (infarct border zone, area of slow conduction). Transmural depolarization must be facilitated using red light-sensitive channelrhodopsin variants such as the novel Chrimson mutants (Mager et al., 2018; Oda et al., 2018) because of the deeper tissue penetration. Also longer lasting Channelrhodopsin variants with 200–500 ms deactivation kinetics could be envisioned in which one or two brief light pulses would result in longer depolarization for low light energy conduction block and optogenetic defibrillation.

### Clinical Perspectives of Implantable Optogenetic Defibrillators

Since publication of the first landmark-trials in the early 2000s (Moss et al., 2002; Bardy et al., 2005), implantation of an ICD has been a cornerstone in the treatment of patients with high risk for ventricular arrhythmia due to heart failure (Ponikowski et al., 2016), cardiac channelopathies, or previously survived sudden cardiac arrest (Priori et al., 2015). Upon detection of a potentially life-threatening ventricular arrhythmia, ICDs apply antitachycardia pacing protocols and subsequently high energy electrical shocks (up to 40 J) to terminate the arrhythmia. Electrical shocks are painful due to stimulation of nerve fibers and direct excitation of skeletal muscles and even low energy shocks for internal cardioversion of AF require sedation (Murgatroyd et al., 1995). Thus, inappropriate electrical shocks, which occur in 4–8% of patients, for example, due to false detection of supraventricular tachycardia (Tan et al., 2014; Kober et al., 2016), have a profound impact on the quality of life including anxiety, depression, and posttraumatic stress syndrome (Kamphuis et al., 2003). Furthermore, sub-studies of the SCD-HEFT trial (Poole et al., 2008) as well as a comparison of successful antitachycardia pacing with electrical shocks for arrhythmia termination (Sweeney et al., 2010) clearly showed increased mortality in patients receiving inappropriate or appropriate ICD shocks. Implanted devices for early AF detection and termination with low energy electrical shocks have been clinically evaluated but were not tolerated because of pain during cardioversion (Geller et al., 2003).

Using specific virus capsids (Zacchigna et al., 2014) or promoters (Werfel et al., 2014) for selective expression of optogenetic actuator proteins in cardiomyocytes would allow in principle painless optogenetic defibrillation and cardioversion. Although yet not proven, it can be anticipated that optogenetic defibrillation by a few seconds of epicardial illumination is less harmful to the heart than electrical shocks. Moreover, sequential light pulses can be applied repetitively, as charging of capacitors to generate ICD electrical shocks is not required. The flexibility of using spatially and temporally shaped light patterns for defibrillation could also be used to minimize secondary pro-arrhythmic effects, which are discussed to be a major cause for electrical defibrillation failure (Charteris and Roth, 2011).

### Technical Challenges Toward Optogenetic Arrhythmia Termination

Before optogenetic therapies can be suggested to patients, the proof-of-concept studies mentioned above have to be verified in preclinical large animal models with human-like anatomy and arrhythmia. Furthermore, long lasting virus-based gene transfer without immunological reactions against viruses or the non-human optogenetic proteins must be established. Toward this aim, we were able to prove that optogenetic defibrillation of VT and cardioversion of AF is also possible in wild-type mouse hearts after systemic injection of AAV (Bruegmann et al., 2016, 2018). Quite surprisingly for the episomal persisting, non-integrating AAV, we found that ChR2 expression was stable for periods of up to 15 months. Optogenetic defibrillation of VT was also confirmed in rats, at least for a period of up to 6 weeks after systemic AAV injection to express a red light-activated ChR2 variant (Nyns et al., 2017). As an alternative to systemic AAV injection, which might infect cells in other organs, gene painting by application of AAV in fibrin clots to the epicardium of the right atrium of rats was shown to result in very localized and highly effective gene transfer sufficient for optogenetic termination of AF (Nyns et al., 2019). Importantly, 4 weeks after gene painting, ~80% cardiomyocytes of the right atrium of immunosuppressed (rapamycin) rats expressed the ChR2 variant compared to only <40% of atrial myocytes >6 months after systemic AAV injection in mice (Bruegmann et al., 2016, 2018). However, long-term stable and transmural ChR2 expression in large animals without immunosuppression remains to be proven. Furthermore, because of the thick left ventricular wall of humans, it is questionable if epicardial gene painting results in sufficient transmural gene expression for optogenetic termination of VT/VF or if systemic or intracoronary infusion of AAV is better suited in this case.

Finally, sufficient transmural illumination must be achieved, e.g., by injectable cellular scale optoelectronics (Kim et al., 2013; Montgomery et al., 2015), LEDs in flexible biocompatible membranes (Xu et al., 2014), or μLED arrays (Gossler et al., 2014). Combining illumination systems with radio-frequency energy harvesters (Park et al., 2015) or with batteries will allow fully implantable illumination devices for chronic optogenetic stimulation of hearts *in vivo*. Recently, a hybrid system for automated AF detection and optogenetic cardioversion in anesthetized rats was described combining surface ECG leads, an external cardiac rhythm monitor, and an implanted atrial LED with a PDMS light guide (Nyns et al., 2019). Such an approach could be extended toward mechanistic investigations of AF-induced fibrotic remodeling of the atria *in vivo* (“AF begets AF”) using fully implantable miniaturized bio-optoelectronic devices for closed-loop optogenetic control in freely moving rats *in vivo* (Mickle et al., 2019).

### Patients Suited for Optogenetic Arrhythmia Termination

Most likely, first patients to benefit from painless and gentle optogenetic defibrillation would be those with recurrent episodes of electrical storm. Electrical storm is defined by three or more sustained episodes of VT/VF with appropriate ICD therapies within 24 h, and the incidence is ranging from 4 to 28% in ICD patients (Huang and Traub, 2008). Mortality is high, and therapeutic options are very limited including interventional catheter ablation from the endocardial or the epicardial side. VT recurrence rates following ablation of sustained VT are high, especially in patients with non-ischemic dilated cardiomyopathy (62 ± 4%) compared to those with ischemic cardiomyopathy (46 ± 4%, median follow-up of 6 years) (Kumar et al., 2016). Patients with ischemic cardiomyopathy typically have a more clearly defined subendocardial or transmural scar, which can be identified as anatomical substrate for re-entry mechanism and thus can be well targeted by endocardial ablation. Patients with non-ischemic dilated cardiomyopathy, however, often have a diffuse mid-myocardial and epicardial fibrosis and frequently require an epicardial ablation procedure (Kumar et al., 2016). Similar to the discussed transmural depolarization for optogenetic defibrillation, the generation of transmural lesions is a key factor for effective ablation, but this cannot always be achieved in the thick ventricular myocardium. In such patients, optogenetic defibrillation would be advantageous, given that transmural depolarization could be achieved with red-shifted optogenetics (see above).

In summary, patients suffering from frequent appropriate and inappropriate ICD shocks despite optimal medical therapy or with ineffective ablation due to diffuse fibrosis from non-ischemic dilated cardiomyopathy could benefit from implantable optogenetic defibrillation devices. Furthermore, an implantable optogenetic “atrioverter” to terminate AF on-demand might be useful to prevent or even revert AF-induced fibrotic remodeling of the atria (“AF begets AF”) (Wijffels et al., 1995).

## Author Contributions

PS and TBr planned the manuscript. PS, MF, TBe, and TBr wrote the manuscript.

### Conflict of Interest Statement

The authors declare that the research was conducted in the absence of any commercial or financial relationships that could be construed as a potential conflict of interest.
